# Pyrosequencing Unveils Cystic Fibrosis Lung Microbiome Differences Associated with a Severe Lung Function Decline

**DOI:** 10.1371/journal.pone.0156807

**Published:** 2016-06-29

**Authors:** Giovanni Bacci, Patrizia Paganin, Loredana Lopez, Chiara Vanni, Claudia Dalmastri, Cristina Cantale, Loretta Daddiego, Gaetano Perrotta, Daniela Dolce, Patrizia Morelli, Vanessa Tuccio, Alessandra De Alessandri, Ersilia Vita Fiscarelli, Giovanni Taccetti, Vincenzina Lucidi, Annamaria Bevivino, Alessio Mengoni

**Affiliations:** 1 Department of Biology, University of Florence, Florence, Italy; 2 Department for Sustainability of Production and Territorial Systems, Biotechnologies and Agro-Industry Division, ENEA Casaccia Research Center, Rome, Italy; 3 Department of Energy Technologies, Bioenergy, Biorefinery and Green Chemistry Division, ENEA Trisaia Research Center, Rotondella (MT), Italy; 4 Department of Pediatrics, Cystic Fibrosis Center, Meyer Hospital, Florence, Italy; 5 Department of Pediatrics, Cystic Fibrosis Center, G. Gaslini Institute, Genoa, Italy; 6 Cystic Fibrosis Microbiology and Cystic Fibrosis Center, Children's Hospital and Research Institute Bambino Gesù, Rome, Italy; University Tuebingen, GERMANY

## Abstract

Chronic airway infection is a hallmark feature of cystic fibrosis (CF) disease. In the present study, sputum samples from CF patients were collected and characterized by 16S rRNA gene-targeted approach, to assess how lung microbiota composition changes following a severe decline in lung function. In particular, we compared the airway microbiota of two groups of patients with CF, i.e. patients with a substantial decline in their lung function (SD) and patients with a stable lung function (S). The two groups showed a different bacterial composition, with SD patients reporting a more heterogeneous community than the S ones. *Pseudomonas* was the dominant genus in both S and SD patients followed by *Staphylococcus* and *Prevotella*. Other than the classical CF pathogens and the most commonly identified non-classical genera in CF, we found the presence of the unusual anaerobic genus *Sneathia*. Moreover, the oligotyping analysis revealed the presence of other minor genera described in CF, highlighting the polymicrobial nature of CF infection. Finally, the analysis of correlation and anti-correlation networks showed the presence of antagonism and ecological independence between members of *Pseudomonas* genus and the rest of CF airways microbiota, with S patients showing a more interconnected community in S patients than in SD ones. This population structure suggests a higher resilience of S microbiota with respect to SD, which in turn may hinder the potential adverse impact of aggressive pathogens (e.g. *Pseudomonas*). In conclusion, our findings shed a new light on CF airway microbiota ecology, improving current knowledge about its composition and polymicrobial interactions in patients with CF.

## Introduction

Despite recent improvements in cystic fibrosis (CF) treatments, some individuals with CF still experience a rapid progression of lung disease, which usually leads to irreversible morbidity and mortality. Bacterial airway infections in CF patients are currently monitored through routine microbiology procedures and cures are mainly based on culturable microbial pathogens colonizing CF airways [1]. Unfortunately, the selection and administration of antimicrobial therapies based on the *in vitro* susceptibility of classic CF pathogens are not necessarily connected with clinical outcomes [2]. In the last years, there has been an increasing awareness that airway colonization is not achieved by a single strain or species but from a complex mixture of microorganisms [3]. Indeed, CF airway microbiota has been shown to be significantly more complex than initially considered [4–7]. Recent works using culture-independent identification methods have revealed that CF patients harbor a vast array of bacterial species, not previously identified and suspected to be involved in CF progression [8–10].

Our understanding of this microbial “black box” has evolved thanks to the development of next-generation sequencing technologies and bioinformatics tools capable of providing new insights into airway microbial communities [11]. Recently, it was proposed that microbial lung community might be considered as a unique, distinct pathogenic entity, whose impact on the host may be greater than the combined effects of its individual component species alone [12]. Even if these findings have drastically altered our understanding of CF lung disease [13,14], their translation into clinical improvements remains a substantial obstacle for enhancing the quality of care [15]. Despite that, a detailed perspective focused on the polymicrobial nature of CF infections would permit to unravel bacterial dynamics providing biomarkers of disease progression, as well as novel bacterial targets for antibiotic treatment [16,15].

Until now, many efforts have been made to expand our understanding of the microbial ecology of the CF airways and new insight into the impact of antibiotic treatment, patient age increasing, and periodic pulmonary exacerbation on CF microbiology have been obtained [8,14,17,18]. Furthermore, a correlation between decreasing diversity of the airways bacterial community and advancing CF lung disease has been found [8] suggesting that reduced diversity may play a role in CF lung disease progression [19]. However, it is still not well clear how the airway microbiota composition changes following a severe decline in lung function, as evidenced by a clinically important drop in FEV_1_ (i.e., with a decrease of 5% or more from baseline values). CF airway infection is viewed as an ecosystem where a *climax* community dominates during relatively stable periods [3]. This ecosystem is dominated by stable populations that are well-adapted to their particular biological niche. A sharp decline in lung function can alter this stable community, possibly shifting it to an alternative state. Our preliminary study, performed by culture-based and culture-independent methods, has provided first insights into relationships between the airway microbiota structure and severe lung function decline [20]. In particular, we identified significant changes in bacterial community diversity when stable (S) and substantial decliner (SD) patients were compared and among patients with different lung disease status (normal/mild, moderate and severe) suggesting that patients with a moderate lung disease (FEV_1_ ranging from 40% to 69%) have experienced changes in their airway bacterial communities [20]. However, the lower discrimination power of molecular methods previously used did not permit to clearly address the CF airway microbiota associated with a severe lung function decline. So, in this study, we employed a deep-sequencing approach based on the 16S rRNA gene-targeted analysis to provide a more-in-depth investigation of the airway microbiota of CF patients with stable lung function (S) versus that detected in patients with a sharp decline in lung function (SD). We considered a subset of previously investigated patients (a total of 52 patients) [20] focusing our attention on S and SD patients only, without considering the differences among the different pulmonary physiopathological status (normal/mild, moderate and severe). New insights into bacterial community features such as biological diversity, taxa composition and interaction among taxa were given.

## Materials and Methods

### Ethics Statement

Protocols for the collection and use of sputum samples from cystic fibrosis patients, and for the procedure of written informed consent were approved by the Ethics Committee of Bambino Gesù Children's Hospital (Rome, Italy), Cystic Fibrosis Center, Meyer Children's Hospital (Florence, Italy) and Giannina Gaslini Children's Hospital (Genoa University, Genoa, Italy) (Prot. N. 681 CM of November 2, 2012; Prot. N. 85 of February 27, 2014; Prot. N. FCC 2012 Partner 4-IGG of September 18, 2012), as previously stated [20]. Informed written consent was obtained from all subjects aged 18 years and over and from parents of all subjects under 18 years of age before enrollment in the study. All sputum specimens were produced voluntarily. The study protocol was in agreement with the *Guidelines of the European Convention on Human Rights and Biomedicine for Research in Children* and to those of the *Ethics Committees of Bambino Gesù*, *Meyer and Giannina Gaslini Hospitals*. All measures were taken to ensure patient data protection and confidentiality.

### Clinical characteristics of patients

Fifty-two patients with CF (aged 8–59 years) who regularly attended the three Italian CF Centers were included in this study. Patients were recruited between September 2012 and April 2013 as follows: Bambino Gesù Children's Hospital (Rome, Italy) (n = 29), Giannina Gaslini Children's Hospital (Genoa, Italy) (n = 14) and Meyer Children's Hospital (Florence, Italy) (n = 9). Patients older than six years of age, who had been diagnosed with CF according to the published Guidelines [21], were treated according to current standards of care [22]. Patients were seen in the CF Centres at least four times per year on a regular basis [1]. At each visit, clinical data collection and microbiological status (colonizing germs) were performed [1]. All patients were clinically stable, without any pulmonary exacerbation (as defined by a cluster of symptoms and signs as previously indicated [23,24]) and antibiotic therapy (*i*.*v*. or oral) in the previous four weeks before specimen collection. A subset of a previously reported [20] cohort of patients was selected. These patients were classified in our previous work [20] into two groups according to their annual rate of FEV_1_ decline by measuring the difference between the best FEV_1_% registered within the previous year and the best FEV_1_% registered two-years before specimen collection. In total, 29 subjects with a rate decline lower than 1.5% were flagged as “stables” (S), whereas 23 subjects with a rate decline higher than 5%, were flagged as “substantial decliners” (SD). Each FEV_1_ value was the average of three repeated measurements obtained 2–3 minutes from each other. FEV_1_ values were measured according to the American Thoracic Society (ATS) and the European Respiratory Society (ERS) standards [25]. The overall description of the patient dataset is reported in Table 1.

**Table 1:** Demographic and clinical characteristics of patients enrolled in the study.

Characteristics	All Patients	Stables (S)	Severe-decliners (SD)
Enrolled CF patients	(n = 52)	(n = 29)	(n = 23)
Sex (n)	23 male	16 male	7 male
	29 female	13 female	16 female
*CFTR* genotype, n (%)			
F508del/F508del	15 (29%)	8 (28%)	7 (30%)
F508del/other	22 (42%)	12 (41%)	10 (44%)
Other/other	15 (29%)	9 (31%)	6 (26%)
Mean age ±SD	27 ± 12	29 ±12	25 ±11
Mean value of FEV_1_% ±SD	63 ± 24	66 ± 29	55 ± 19
Disease stage categories, n (%)			
Normal/mild (FEV_1_% > 70)	23 (44%)	13 (45%)	10 (44%)
Moderate (70 ≥ FEV_1_% ≥ 40)	17 (33%)	10 (34%)	7 (30%)
Severe (FEV_1_% < 40)	12 (23%)	6 (21%)	6 (26%)

### Sample processing and DNA extraction

Spontaneously expectorated sputum (SES) was used in this study, as it represents by far the most widely used sample in productive patients [26]. Samples processing was performed as previously described [20]. About 400 μl aliquots of frozen sputum were subjected to genomic DNA extraction using the commercially available Kit QIAamp DNA Mini Kit. Sample aliquots were spun at 10,000×g to pellet cellular material. After removal of the supernatant, cell pellets were resuspended in 180 μl of the appropriate enzyme solution (20 mg/ml lysozyme; 20 mM Tris·HCl, pH 8.0; 2 mM EDTA; 1.2% Triton), incubated for 30 min at 37°C and then processed according to the manufacturer’s protocol. Quantity and purity of extracted DNA were checked by NanoDrop (NanoDrop Technologies, USA), PicoGreen fluorescent assay (Life Technologies), and gel electrophoresis.

### DNA amplification and sequencing

DNA samples were subjected to 16S rRNA gene amplification. Primers and barcodes were designed according to the Human Microbiome Project Consortium (HMP) (http://www.hmpdacc.org/tools_protocols/tools_protocols.php). The V3, V4, and V5 hypervariable regions of the 16S rRNA gene were amplified using primers 357F (*5′-CCTACGGGAGGCAGCAG-3′*) and 926R (*5′-CCGTCAATTCMTTTRAGT-3′*) modified with the addition of the 454 FLX-titanium adaptors B and A, respectively. Unique 7-nucleotide barcode sequences (MID 1–18) were added to the A adaptor as reported in S1 Table. PCR amplification was performed on 2 μl of DNA template in a total volume of 25 μl containing 1× AccuPrime Buffer II (Life Technologies), 10 μM of 357F fusion-primer, 10 μM of 926F fusion-primer and 0.03 U/μl AccuPrime High-Fidelity *Taq* DNA polymerase (Life Technologies). PCR reactions were heated at 95°C for 2 min followed by 30 cycles of 95°C for 20 seconds, 55°C for 30 seconds, and 72°C for 5 minutes. All reactions were prepared in a sterile PCR hood. Three independent PCR reactions were performed for each DNA-template. Negative control reactions were also performed. Each replicate reaction was examined by electrophoresis on 1.5% agarose gel. PCR amplicons were cleaned using the Agencourt AMPure XP Beads according to the manufacturer’s specifications (Beckman Coulter). To ensure removal of primers and any non-specific amplicons, purified amplicon libraries were analyzed using the Agilent Bioanalyzer 2100 employing the Agilent DNA 1000 Kit. The Quant-iT PicoGreen dsDNA fluorescent assay Kit (Life Technologies) was employed to establish the concentration of the purified amplicons. Three independent purified and quantified PCR reactions were pooled in equimolar proportion foe each sample. Pools were quantified using KAPA Library Quantification Kits (KAPA Biosystems) to determine the number of amplifiable molecules in the libraries. Emulsion PCR, emulsion breaking, and amplicon pyrosequencing were performed applying the 454 GS FLX+ chemistry following supplier protocols (454 Life Sciences Roche Corporation). The GS FLX+ software 2.9 version was used for sequencing and the pipeline 3 for long amplicon was used to data processing (454 Life Sciences Roche Corporation). Sequence files have been uploaded to the NCBI Sequence Read Archive (SRA) with the accession number: PRJNA290694.

### Sequence processing and data generation

Amplicon sequences were demultiplexed using Mothur [27], discarding those with mismatches in the barcode, primers, linkers or spacers. A first quality check step was performed using StreamingTrim [28] to remove ambiguous base calls (Phred’s quality score < 25) and convert sequences into FASTQ format. Filtered sequences were processed following the UPARSE pipeline included in the USEARCH package (version 7.0.1090) [29]. In particular, sequences were trimmed to a fixed length of 250 base-pairs (bp) through the “fastq_trunclen” command. Chimeras were removed from pooled sequences with the “uchime_denovo” algorithm, and Operational Taxonomic Units (OTUs) were generated with an identity cutoff of 97% (which is approximately close to the taxonomic level of species according to [30]. SINA standalone classifier was used for taxonomic assignment of representative sequences in combination with the most recent version of SILVA non-redundant database available (SSU Ref NR 99, release 115). Genera were categorized as aerobic/anaerobic and pathogenic/non-pathogenic as reported in [10,31]. OTU oligotyping [32] was performed following the procedures reported in http://merenlab.org/2013/11/04/oligotyping-best-practices/ with a minimum substantive abundance criterion (M) of 50. We considered an OTU fully resolved when all its olygotypes had a purity index of at least 90%. For each oligotype, the first 5 BLAST best hits [33] corresponding to a defined taxonomy with 100% identity and 100% coverage were reported.

### Statistical analyses

To analyze FEV_1_ decline between patient groups, mixed-effect models with random intercept and slope were used as data contained repeated measures of the same variable (FEV_1_ index) for each unit (patient) in the dataset. Shapiro-Wilk test was used to determine to determine if data were normally distributed. Thus different linear mixed-effect models were compared through the likelihood ratio test choosing the one which best fitted our data; for additional information about fitted models see S1 Appendix and S1 Fig. Mixed-effect models were fitted and compared in R version 3.2.5 using the lme4 [34] package version 1.1.

Bacterial diversity was inspected through Shannon, Chao 1, Evenness and Richness indices (the latter expressed as the count of OTUs observed in each sample) on both rarefied and non-rarefied counts. Since differences between rarefied and non-rarefied values were really low (less than 0.01%, data not shown), non-rarefied values were reported. All indices were computed with the R [35] package vegan [36]. The percentage of coverage was calculated by Good's estimator [37] using the formula: [1—(n/N)] × 100, where n is the number of sequences found once in a sample (singletons), and N is the total number of sequences in that sample. The Evenness index was calculated using the formula E = S/log(R), where S is the Shannon diversity index and R is the number of OTUs in the sample (the Richness). Bacterial communities from different groups of patients were compared using Multivariate Analysis of Variance (MANOVA) with Pillai-Bartlett statistic. Single OTUs and oligotypes were compared through the Analysis of Variance (ANOVA) in combination with the Tukey's honest significant difference (HSD) test. Canonical Correlation Analysis (CCA) was performed using the log transformed OTUs abundance. All statistical analyses and graphical representations were carried out using the R software.

### Network construction

Co-occurrence networks were constructed following previously reported methods [38,39] Undirected graphs reporting the correlation between OTUs were produced. Each node corresponds to a different OTU, whereas each edge represents a significant (p < 0.05) Spearman’s correlation. The diameter of the nodes was directly proportional to the OTU occurrence in the dataset whereas the width of the edges was directly proportional to the Spearman’s correlation index. Produced graphs were arranged using the Fruchterman Reingold algorithm [40]. All statistical analyses were carried out in the R environment using the igraph package for network generation [41].

## Results

### Airways bacterial community structure in S and SD patients

Sputum DNA from 52 specimens, obtained during routine medical care, in accordance with the ethical Guidelines, was analyzed by pyrosequencing of the V3–V5 hypervariable region of the 16S rRNA gene. A total of 201903 reads were generated from these samples with an average length of 541 bp. After demultiplexing, quality control, chimera detection, and length filtering, 158107 high-quality sequences of 250 bp each were collected with an average of 3040 sequences per sample (ranging from 880 to 6381). The number of sequences and OTUs detected for each patient has been reported in S2 Table.

**Fig 1:**
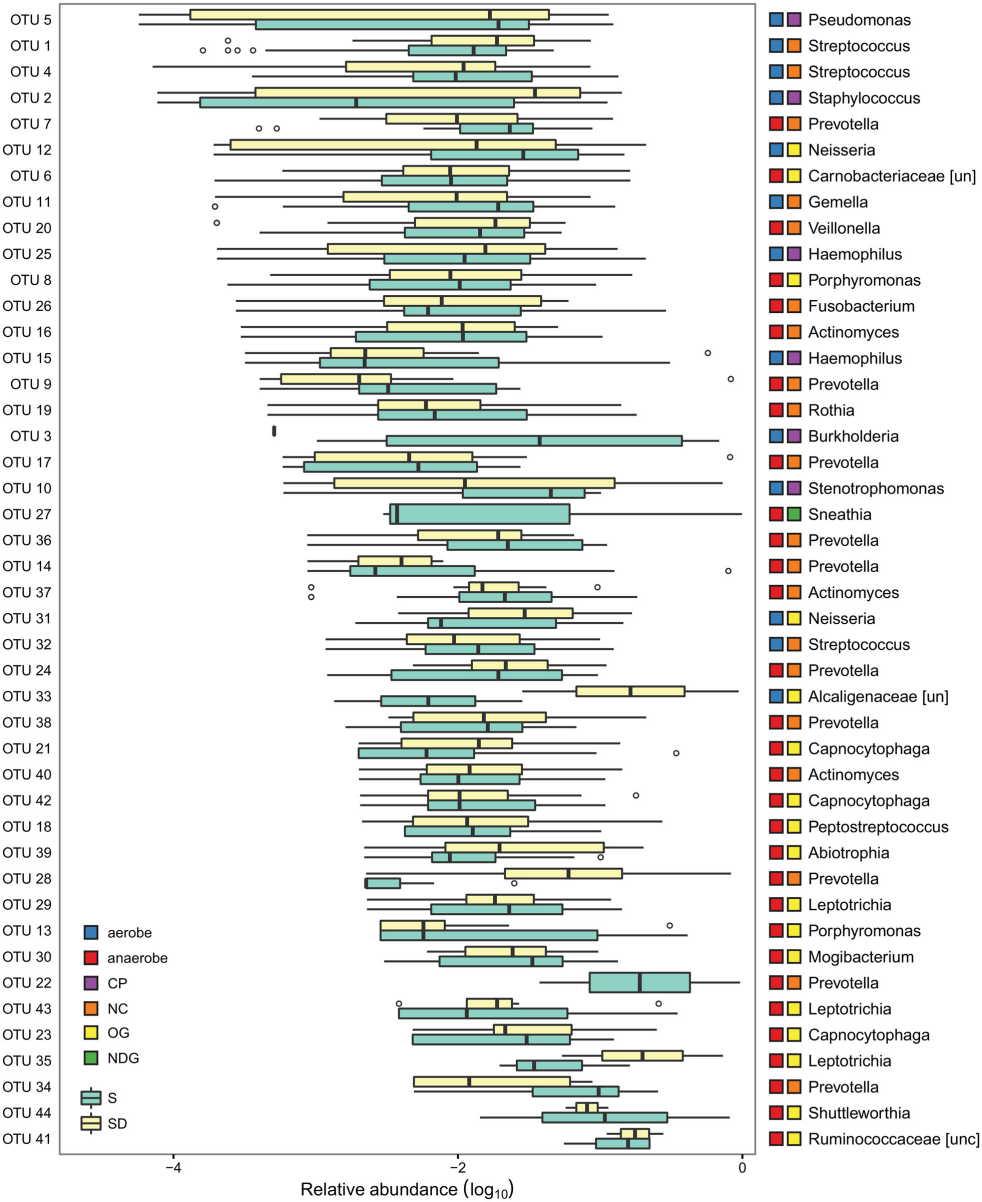
Relative abundance of sputum microbiota among patients. Relative OTUs abundance was computed dividing the number of 16S sequences assigned to each OTU by the total number of sequences obtained for each sample. Boxes denote the interquartile range (IQR) between the 25^th^ and the 75^th^ percentile (first and third quartiles), whereas the inner line represents the median. Whiskers represent the lowest and highest values within 1.5 times IQR from the first and third quartiles Outliers were reported using white circles. CP: common CF pathogens; NC: non-classical but commonly identified genera in CF; OG: other genera in CF; NDG: not yet described genera in CF; S: stable patients; SD: severe decliner patients; [un] unclassified; [unc] uncultured.

To investigate the depth of our 16SrRNA amplicon libraries, rarefaction curves were computed on OTU assignments. Most rarefaction curves approached asymptote (S2 Fig), suggesting that our sampling efforts were able to represent bacterial community assemblages of all patients. Moreover, Good's estimator reported a satisfactory coverage (higher than 99% for all 52 samples, S2 Table). Forty-four OTUs (based on 97% sequence similarity) were detected with each sample ranging from 10 to 35 OTUs (S2 Table) (median = 25). Twenty-two genera were detected with a high intra- and inter- groups variance (Fig 1). All the genera recovered so far from the respiratory tracts of patients with CF were detected, with the only exception of the anaerobic genus *Sneathia*. The analysis of variance of diversity indices (Shannon index, Chao1 and Evenness) based on OTU counts, showed that different conditions did not alter the bacterial diversity of the lung microbiota which remained almost constant in each group (S3 Fig).

OTU 5 was the most abundant (35052 sequences), and it was the only one classified as *Pseudomonas*. Moreover, *Pseudomonas* was the most abundant genus in 33% of patients, followed by *Staphylococcus* (OTU 2, 12990 reads in total and the most abundant genus in 19% of patients). Ten OTUs were classified as *Prevotella*, which was found in 96% of the subjects with a total of 16353 sequences (ranging from 0.02% to 74.49% of the total sequences in each sample). In one sample, *Prevotella* was the dominant genus followed by *Staphylococcus* (8.5% of sequences). Two OTUs could not be identified at the genus level, and they were reported using their family attribution with the unclassified flag “un” (*Carnobacteriaceae*, 5143 sequences in 43 samples and *Alcaligenaceae*, 741 sequences in 4 samples). Only one OTU was classified as an “unculturable organism belonging to the *Ruminococcaceae* family” (18 sequences in 6 samples). Interestingly, when considering genus abundance, *Pseudomonas* showed a significant negative correlation with microbial diversity in both groups of patients (Shannon index: r < -0.4, p < 0.05; Chao 1 index: r < -0.4, p < 0.05; Richness index: r < -0.5, p < 0.01, Spearman's rank correlation coefficient for both S and SD group). The abundance of aerobic and anaerobic genera did not show significant differences between the two groups of patients. Similarly, the abundance of the most common CF pathogens did not seem to change in S and SD groups (Wilcoxon signed-rank test: p > 0.05 for all contrasts).

**Fig 2:**
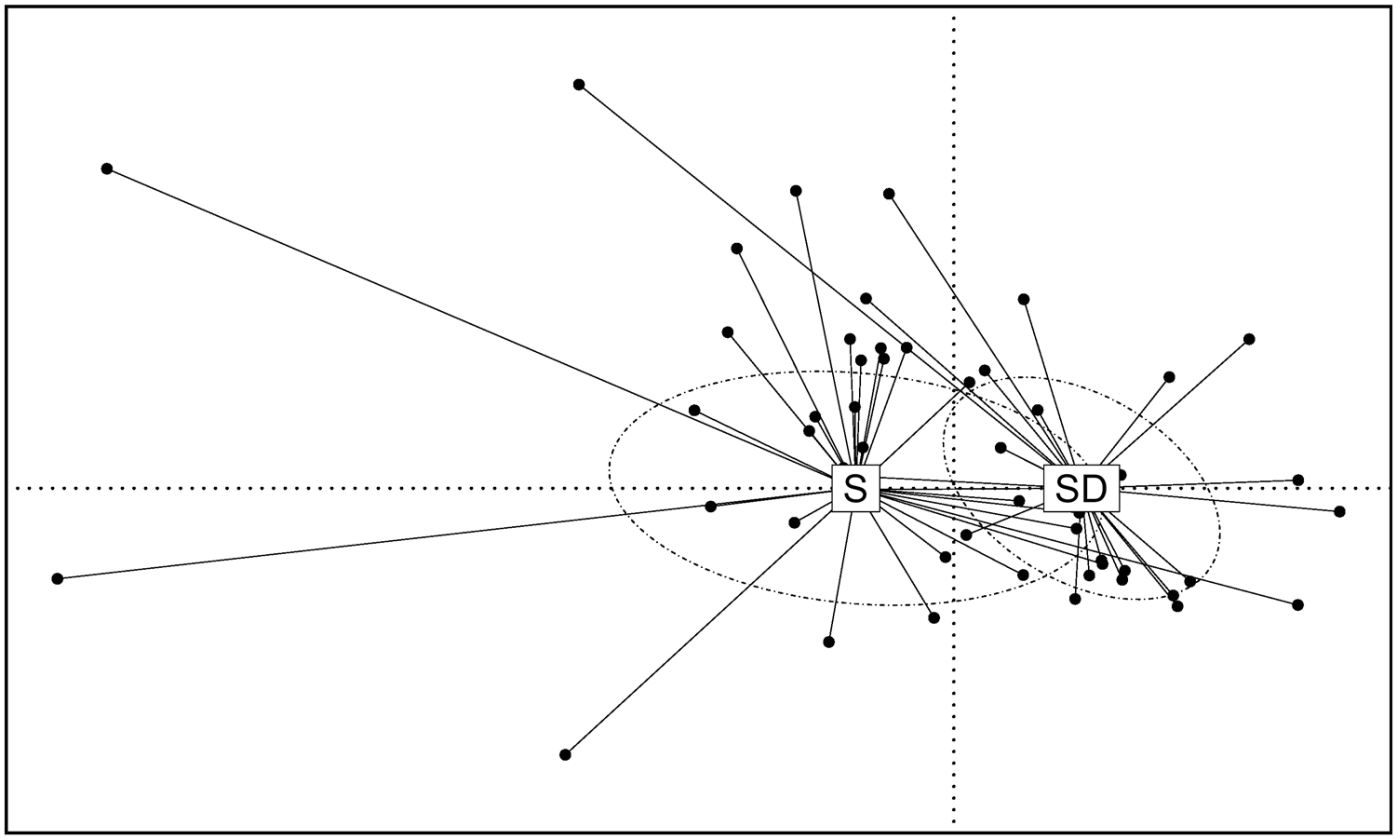
Canonical Correlation analysis based on the log-transformed abundance of 44 OTUs. Patient conditions were used as constraining variable. Two first components (CCA1 and CA1) were plotted accounting for 15.6% of overall inertia of the data set. Individuals (represented by points) were clustered, and centroids were computed for each group. Ellipses were drawn using the standard deviation of points belonging to the same cluster with a confidence limit of 95%.

The relationship between global bacterial community composition and patient groups was then inspected using CCA and two-way MANOVA. The CCA analysis (which explained the 15.7% of the overall inertia, CCA1: 1.6%; CA1: 14.1%), indicated a slight overlap between patient groups (Fig 2). However, a significant correlation was found between the ordination analysis and patients’ conditions (Environmental fitting based on 1 000 permutations: p < 0.01).

**Fig 3:**
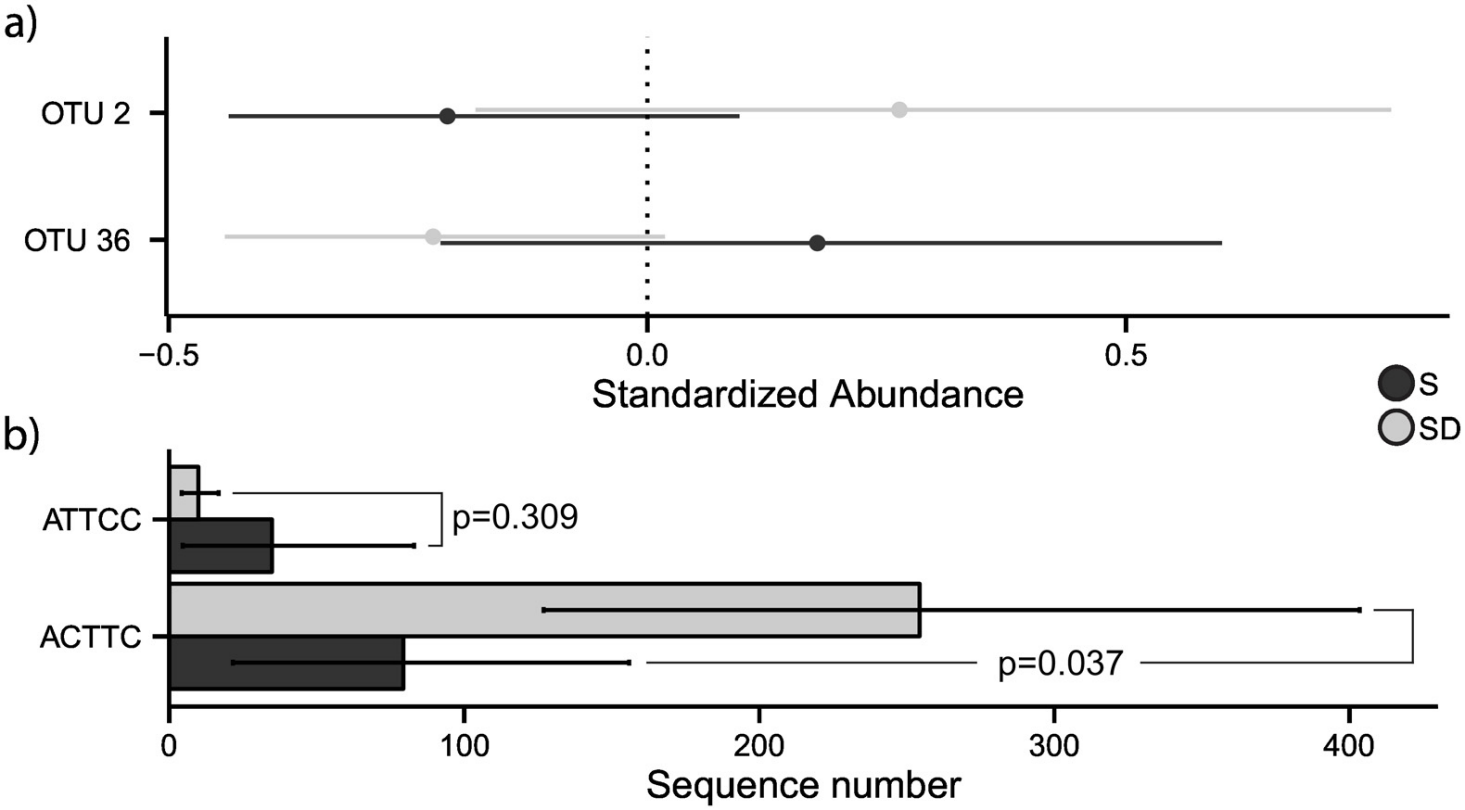
Differences in microbial community distribution between stable and severe decliner groups. Bacterial taxonomic profiling revealed differences between stable and severe decliner communities. a) Standardized abundances of two OTUs (OTU 2 and 36) showing a different distribution between patient groups. b) Differences between the two most represented oligotypes identified for OTU 2. Standardize abundances were calculated as: [x - mean(x)]/sd(x), where "sd" is the standard deviation and “mean” is the mean value. As a result, all OTUs have equal means and standard deviations (0 and 1, respectively) but different ranges. Reported p values were obtained with a Wilcoxon signed-rank test.

The presence of a microbiota signature of patients groups was also supported by two-way MANOVA (Pillais’ Trace = 0.99, p < 0.05; S3 Table). In particular, two OTUs (OTU 2, classified as *Staphylococcus* and OTU 36, classified as *Prevotella*) showed a different distribution between groups of patients (ANOVA: p < 0.05; Tukey HSD: p < 0.05; Fig 3a).

OTUs were further characterized by oligotype analysis aiming to detect the presence of different species/strains within the same genus. For OTU 2 (classified as *Staphylococcus*), by using five entropy locations (68^th^, 73^th^, 80^th^, 175^th^ and 178^th^ position), we identified three oligotypes: ACTTC (8 218 reads in 36 subjects), ATTCC (1278 reads in 33 subjects) and ATTCT (104 reads in one subject) (Table 2).

**Table 2:** BLAST analysis of OTU 2 and OTU 36 oligotypes.

OTU	Oligotype (sequences)	BLAST hit (16S rRNA gene)	Accession
OTU 2	ACTTC (8218)	*Staphylococcus aureus*	LN929739
		*Staphylococcus aureus*	LN929738
		*Staphylococcus aureus*	LN929737
		*Staphylococcus sp*. *RB53*	KT216039
		*Staphylococcus sp*. *RB52*	KT216038
	ATTCC (1278)	*Staphylococcus epidermidis*	KT184898
		*Staphylococcus hominis*	KP240988
		Staphylococcaceae bacterium	JX064866
		*Bacterium RA7P1*	GU366198
		*Staphylococcus xylosus*	JQ726638
	ATTCT (104)	*Staphylococcus sp*. *LW-36*	KR258784
		*Staphylococcus sp*. *MB30*	KJ531648
		*Staphylococcus epidermidis*	KT184898
		*Staphylococcus hominis*	KP240988
		Staphylococcaceae bacterium	JX064866
OTU 36	GGT (825)	Uncultured *Prevotella sp*.	JF893616
		*Prevotella pallens*	NR_113121
		*Prevotella pallens*	GU561354
		*Prevotella sp*. oral taxon	GQ422737
		*Prevotella pallens*	NR_026417

Oligotypes are reported with their name followed by the number of assigned sequences between brackets. Only 16S rRNA genes were reported as valid BLAST hits.

The first oligotype (the most abundant) was the only one to show a different distribution between S and SD patients (Wilcoxon signed-rank test: p < 0.05), as reported in Fig 3b. BLAST analysis reported different taxonomic annotations for the three oligotypes, with the ACTTC oligotype matching *Staphylococcus aureus* sequences in the first three BLAST best hits (Table 2). OTU 36 was totally resolved with three entropy locations (12^th^, 196^th^ and 246^th^ positions) accounting for a single olygotype. Its taxonomic attribution was confirmed by BLAST analysis, which reported three different members of *Prevotella* genus (Table 2). In addition to OTU 2 and OTU 36, we performed oligotype analysis on the OTU 5. This OTU was the only one assigned to the *Pseudomonas* genus, which has been correlated negatively with bacterial diversity as reported above. Oligotype analysis produced ten different oligotypes most of which reporting *P*. *aeruginosa* as their first BLAST hit (for additional details see S2 Appendix).

### Network analysis

**Fig 4:**
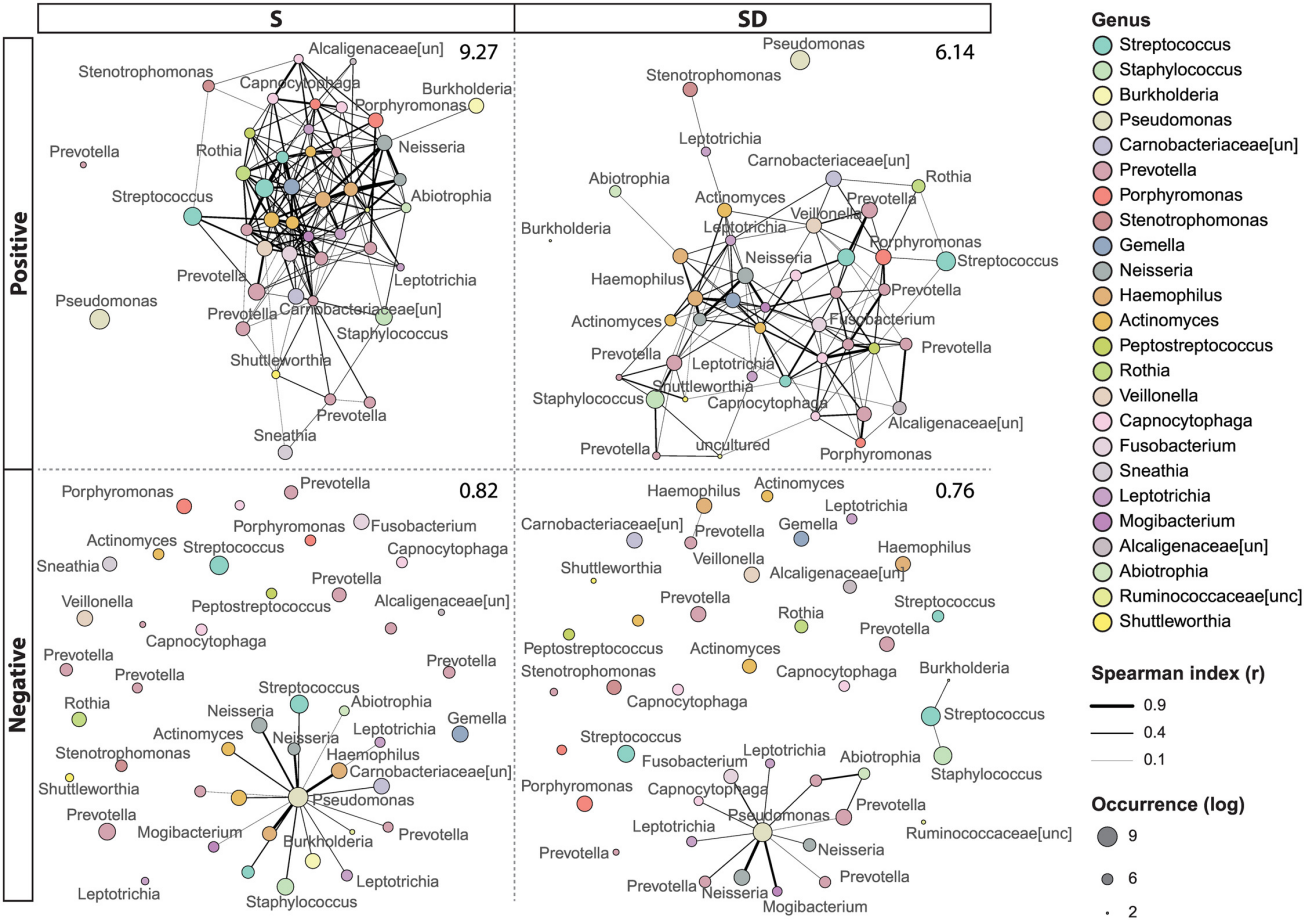
OTU Networks based on correlation analysis. Networks report positive and negative correlations between OTUs found in the airway microbiota of S and SD patients. Node color corresponds to taxonomic assignments, whereas node size reflects the log-transformed abundance. Edge thickness is proportional to the modulus of Spearman's rank correlation coefficient. Average degree values are reported in the upper-right corner of each network.

Aiming to investigate if patients’ groups may reflect ecological niche processes at the airway level that drive coexistence within microbiota, co-occurrence relationships were analyzed. Four networks were produced reporting positive and negative correlations between OTUs detected in S and SD patients (Fig 4).

The positive correlation could indicate cooperative interactions or the presence of common biological functions or ecological niche between taxa. Negative correlations could be indicative of either competitive interactions or non-overlapping ecological niches or processes between taxa [42]. Spearman's rank correlation coefficient obtained in our dataset ranged from 0.37 to 0.81 and from -0.42 to -0.70 in positive and negative networks, respectively. Considering global network properties, positive correlation networks showed a higher number of edges than the negative ones. Interestingly, the networks of SD patients showed lower average degree values in both positive and negative correlation on those of S patients. Finally, *Pseudomonas* showed no positive correlations with any other member of the microbial community of both S and SD patients but represented the node with the highest number of links in the negative correlation networks (with 18 and 11 edges for S and SD group, respectively).

## Discussion

During the last five years, the approach to the study of CF airway infections has changed. The analysis of single pathogens has been overcome by the analysis of lung bacterial community as a whole. Indeed, several studies have shown that bacterial community in CF lung is very heterogeneous and that its variability may be influenced by multiple factors, such as patient age, exacerbations status, antibiotic treatment and pulmonary function decline [5,8,18,43]. Given the importance of lung function in CF patients’ health, it is by extension important to understand the complexity of CF microbiota especially in patients displaying fluctuating levels of lung function, identifying ecological factors associated with higher/lower pulmonary function decline. As we continue to enhance our knowledge of the airway microbiota, these factors and other unanswered questions will guide future research efforts directed towards understanding the complex interactions governing the host–microorganism relationship.

In a previous work [20] we provided first insights into the CF airway microbiota of S and SD patients. By combining culture-based methods and Terminal Restriction Fragment Length Polymorphism (T-RFLP) analysis of amplified 16S rRNA gene from sputum with clinically relevant information, we found that the bacterial community in SD patients had clear differences in alpha diversity parameters. However, due to the limitation of T-RFLP in taxa identification, we did not detail which bacterial taxa mainly contribute to determining such differences between S and SD patients’ airways bacterial communities, nor we did provide a clearer picture regarding the dynamic interactions between bacterial communities. The approach used in this study allowed us to assess exhaustively the bacterial composition in CF samples showing the presence of 22 distinct bacterial genera, with a massive presence of *Pseudomonas* and *Staphylococcus* representatives. In particular, *Staphylococcus aureus* was more abundant in S patients than in SD ones. Interestingly, we were able to identify several members of *Prevotella* genus, which is considered as an emerging CF pathogens [43,44]. Moreover, thanks to the culture-free approach here adopted we were able to detect an unclassified member of *Alcaligenaceae* (a family in the order *Burkholderiales* of the β-*Proteobacteria* [45]) and *Carnobacteriaceae* families along with the unusual anaerobic genus *Sneathia*. Representatives of the first family have been isolated from various clinical samples, including respiratory secretions of CF patients [46]. Among them, *Achromobacter xylosoxidans* represents one of the most important emerging CF pathogens [47]. The recovery of organisms that are not reported by using the routine clinical protocols such as members of the Carnobacteriaceae were previously reported by expanded culture-dependent profiling of CF airway microbiology [48]. Conversely, to the best of our knowledge, members of *Sneathia* have never been associated with CF airways microflora. However, reports of *Sneathia* occurrence in lung transplant are present [49], suggesting that this unusual anaerobic genus could indeed colonize lung tissue. It is worth mentioning that, due to the resolution limit of 16S rRNA based analyses, further taxonomic characterization was not possible for members of these families within our subjects. In general, results obtained allowed us to confirm the value of culture-based methods. However, the most commonly identified non-classical genera in CF, as well as other minor genera described in CF have been detected by pyrosequencing analysis, highlighting the polymicrobial nature of CF infection [7,50], but their relation with lung decline deserves further attention and investigation.

The dynamic balance in the airways microbiota ecosystem depends on interactions among bacterial species as well as between bacteria and the host environment [51]. Until now, microbe-microbe interactions in CF have been evaluated under *in vitro* conditions, focused mainly on the traditional pathogen *P*. *aeruginosa*, and have revealed the interplay among the classical CF pathogens [50]. Applying established ecological principles to the study of CF airway infection has produced important biological and pathophysiological insights into CF airway infection [3]. Network analysis of taxon co-occurrence patterns gives new insight into the structure of complex microbial communities [39] and offers one of the best apparatus available today to tackle the tangled human microbiota [51]. Positive correlation networks revealed widely associated bacterial communities, showing no connections with *Pseudomonas*. A significant negative correlation was found between bacterial diversity and the presence of members of *Pseudomonas* genus. Furthermore, negative correlation networks showed a massive number of negative correlations between *Pseudomonas* and other members of lung microbiota, suggesting a strong antagonistic potential of *Pseudomonas* over the airway microbiota and the other CF pathogens [52]. Interestingly, the network degree values (i.e. the number of interaction each taxon has with the others) is higher in S than in SD group, suggesting that the S airway microbiota could be more resilient to environmental changes (as for instance those due to the spread of a novel opportunistic pathogen, such as *Pseudomonas*). Indeed, a direct link between degree of the correlation network and community resilience has been shown in other systems [53–55]. We can consequently speculate that airway bacterial community, as a whole, can confer a benefit to S patients with respect to the perturbation caused or associated with the spread of an opportunistic pathogen (e.g. *Pseudomonas*). The interactions within the microbiota network can influence the healthy or disease status of the human body. The loss of any interconnections between bacteria belonging to *Pseudomonas* genus and the OTUs co-occurrence offers a piece of concrete evidence to support the role of this important pathogen in CF disease. However, diversity measurements are not sufficient to predict patient status in a cross-sectional study [44]. Indeed, longitudinal studies, following a cohort of patients over time, may help to elucidate the microbial factors that can contribute to their status changes.

In conclusion, we provided an in-depth description of bacterial community composition based on the analysis of 16S rRNA gene sequence at different resolution levels from genera to olygotype characterization. Our findings revealed a different structure and composition of airway microbiota in CF airways of S and SD patients. The severe decline in lung function experienced by CF patients was associated with a lower complexity of microbial community, which in turn could have paved the way for the proliferation of pathogenic bacteria (such as members of *Staphylococcus* genus), whereas the stable lung function condition favours a relatively complex bacterial community. The correlation network analysis showed a decreased number of positive correlations in SD patients microbiota with respect with S ones. In such analysis *Pseudomonas* emerged as the most negatively connected genus, suggesting the presence of a high number of competitive interactions between *Pseudomonas* and the other taxa. A high presence of *Pseudomonas* would consequently reduce the number of other taxa inhabiting the airways bacterial community, and then the overall community diversity. Cross-sectional analysis is, of course, unable to show how community dynamics change, as a result of immune response or treatment. This is why a metagenomic longitudinal analysis is mandatory as it might lead to the identification of predictors of clinical change. The possibility to analyze the meta-community dynamics and to identify signatures for S and SD patients can give us a set of tools to unlock the potential of microbiome-based personalized medicine in major disease areas including CF.

## Supporting Information

S1 Appendix Supplementary Methods. (DOCX)

S2 Appendix Supplementary Results. (DOCX)

S1 Fig FEV_1_ values for each patient in the two years before the enrollment. Enrollment date is reported next to the subject ID whereas the sampling year is reported in each x-axis. Blue lines represent predicted values based on mixed-effect models whereas red crosses were used to mark the best values in each sampling year. The difference between the best FEV_1_ values registered one year and two years before the enrollment was reported with dashed lines.
(PDF)

S2 Fig Rarefaction curves based on the number of OTUs found in each sample. (PDF)

S3 Fig Diversity indices in the two groups of patience observed. Each p-values obtained through the analysis of variance (ANOVA) was reported along with the name of the index analyzed. Boxes denote the interquartile range (IQR) between the 25^th^ and the 75^th^ percentile (first and third quartiles), whereas the inner line represents the median. For a more detailed description about boxes see Fig 1.
(PDF)

S1 Table Primers and barcodes used for 16S rRNA gene amplifications and sequencing. (DOCX)

S2 Table Number of sequences, OTUs, diversity indices and Good’s coverage estimator for all patients enrolled in the study. (DOCX)

S3 Table Multivariate analysis of variance (MANOVA) on OTUs dataset. (DOCX)
