# Correction: Pyrosequencing Unveils Cystic Fibrosis Lung Microbiome Differences Associated with a Severe Lung Function Decline

**DOI:** 10.1371/journal.pone.0160726

**Published:** 2016-08-01

**Authors:** Giovanni Bacci, Patrizia Paganin, Loredana Lopez, Chiara Vanni, Claudia Dalmastri, Cristina Cantale, Loretta Daddiego, Gaetano Perrotta, Daniela Dolce, Patrizia Morelli, Vanessa Tuccio, Alessandra De Alessandri, Ersilia Vita Fiscarelli, Giovanni Taccetti, Vincenzina Lucidi, Annamaria Bevivino, Alessio Mengoni

The affiliation for the sixteenth author is incorrect. Annamaria Bevivino is not affiliated with #1 but with #2 Department for Sustainability of Production and Territorial Systems, Biotechnologies and Agro-Industry Division, ENEA Casaccia Research Center, Rome, Italy.
