# Supplementary material for: Pyrosequencing Unveils Cystic Fibrosis Lung Microbiome Differences Associated with a Severe Lung Function Decline
Source: PLoS One. 2016 Jun 29;11(6):e0156807. doi: 10.1371/journal.pone.0156807 (PMC4927098; doi:10.1371/journal.pone.0156807)
Supplement: S1 Appendix — (DOCX) [file pone.0156807.s001.docx]

**S1 Appendix: Supplementary Methods**

**Linear mixed-effect models:** The FEV_1_ decline was evaluated through linear mixed-effect models using the R package “lme4” as described in the main text. All fitted models were reported using the standard R formula notation. First, the effect of time was evaluated using a basic random intercept model with a different intercept for each patient:

here FEV1, time and ID correspond to the FEV_1_ values, the sampling time and the patient id, respectively. The effect of time was highly significant (p-value << 0.01; likelihood ratio test based on 1000 simulated values), lowering FEV_1_ values by about 3.0% per year in the whole dataset. Next, an extra covariate coding the group (S or SD) was added to the model to test different effects of time in the two groups considered:

FEV1, time and ID were the same as before whereas Group represented the variable coding patient groups. The interaction between time and patient groups was highly significant (χ^2^ = 129.56, p << 0.01), indicating a different decline of the FEV_1_ index in the two groups considered. Finally, a random intercept and slope model was fitted to allow patients to have not only differing intercepts, but even different slopes:

As expected, the random slope effect was significant (χ^2^ = 31.21, p << 0.01), with patients from SD group reporting lower values of FEV_1_ than patients from the S group.
