# Supplementary material for: Pyrosequencing Unveils Cystic Fibrosis Lung Microbiome Differences Associated with a Severe Lung Function Decline
Source: PLoS One. 2016 Jun 29;11(6):e0156807. doi: 10.1371/journal.pone.0156807 (PMC4927098; doi:10.1371/journal.pone.0156807)
Supplement: S2 Appendix — (DOCX) [file pone.0156807.s002.docx]

**S2 Appendix: Supplementary Results**

***Pseudomonas* oligotyping:** we were able to fully resolved OTU 5 using10 high-entropy locations: 1^st^, 3^rd^, 4^th^, 8^th^, 15^th^, 20^th^, 34^th^, 75^th^, 88^th^, 152^th^, 229^th^, 232^th^, 240^th^, 243^th^, 244^th^, 246^th^ and 249^th^ position in the alignment. These produced 10 slightly different oligotype sequences reporting different BLAST annotations. The most abundant olygotype accounted for 24 141 sequences (88.97% of the reads) with 4 out of 5 BLAST best hits belonging to *Pseudomonas aeruginosa* strains and present in all subjects with at least one OTU 5 assignment. Among the other 9 oligotypes, 6 (8.9% of the reads) matched to at least one *Pseudomonas aeruginosa* strain in their first 5 BLAST best hits. None of the detected oligotypes displayed significant differences between stable and severe decliner groups (ANOVA: p values > 0.05 for all contrasts).
