# Supplementary figures and images for: Pyrosequencing Unveils Cystic Fibrosis Lung Microbiome Differences Associated with a Severe Lung Function Decline
Source: PLoS One. 2016 Jun 29;11(6):e0156807. doi: 10.1371/journal.pone.0156807 (PMC4927098; doi:10.1371/journal.pone.0156807)

# Stable Patients

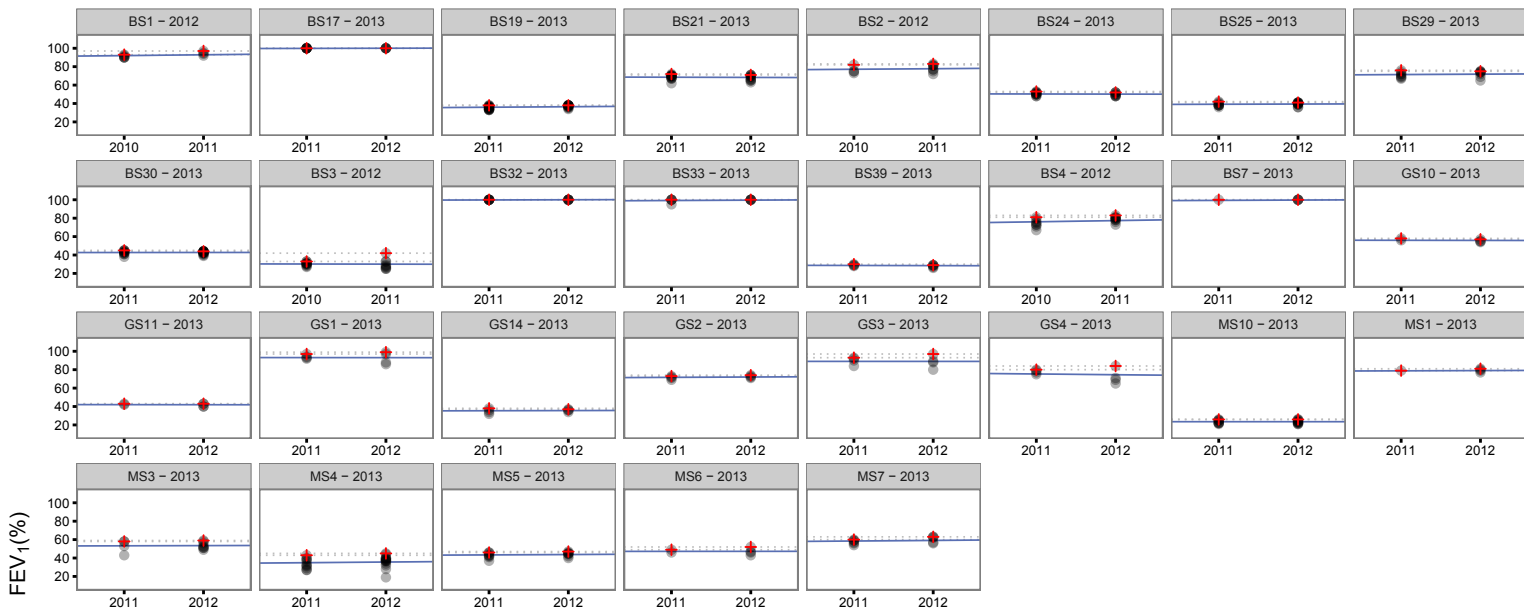

# Severe Decliner Patients

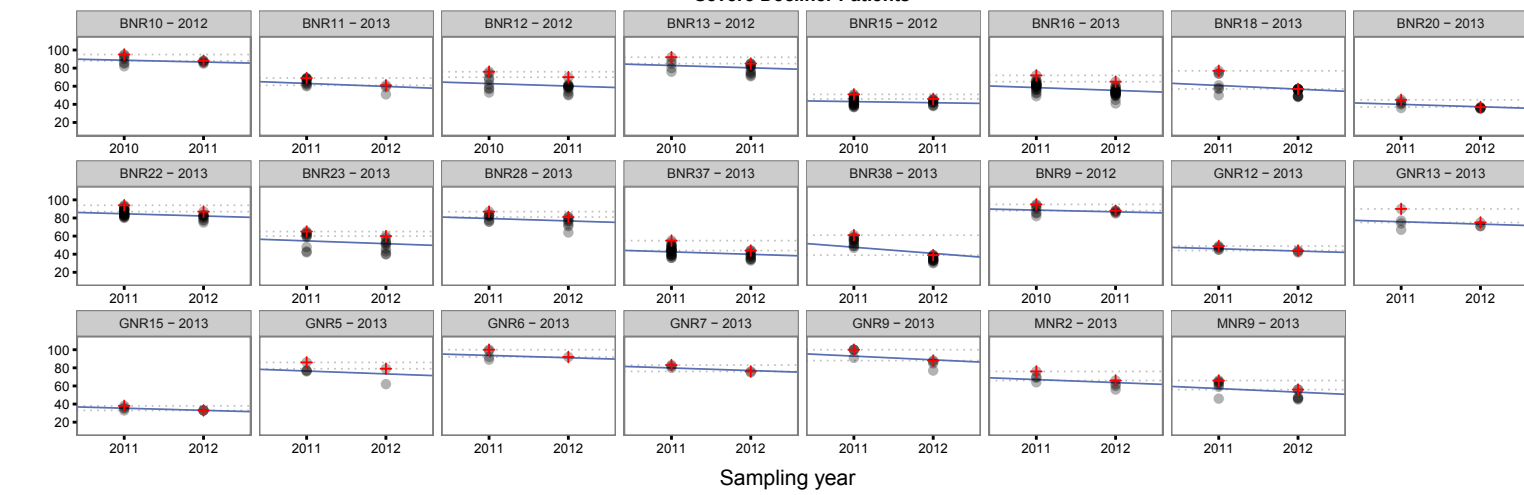

Supplement: S1 Fig — Enrollment date is reported next to the subject ID whereas the sampling year is reported in each x-axis. Blue lines represent predicted values based on mixed-effect models whereas red crosses were used to mark the best values in each sampling year. The difference between the best FEV1 values registered one year and two years before the enrollment was reported with dashed lines. (PDF) [file pone.0156807.s003.pdf]

Number of OTUs

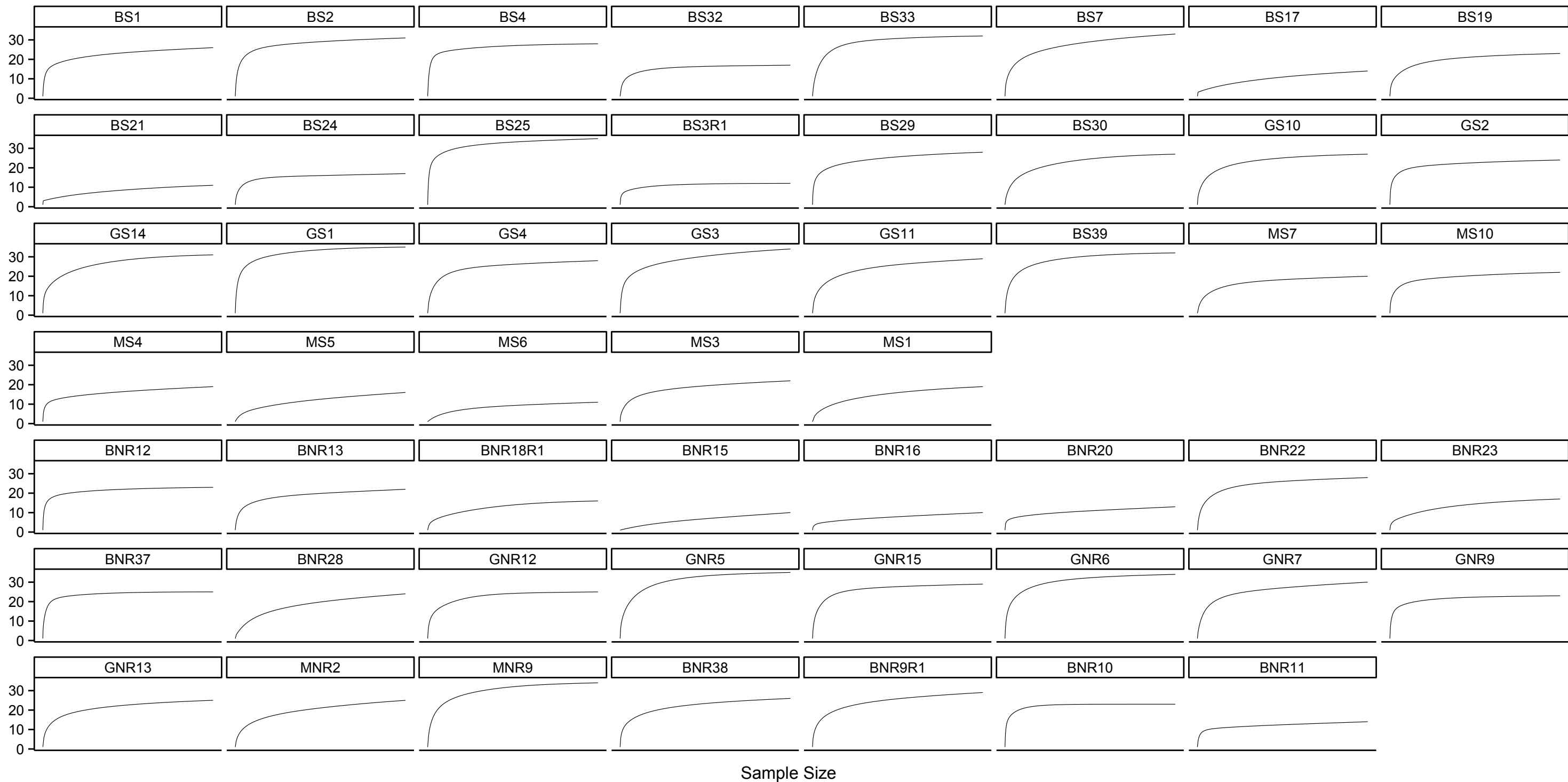

Supplement: S2 Fig — (PDF) [file pone.0156807.s004.pdf]

**Shannon**  
**p = 0.3478**

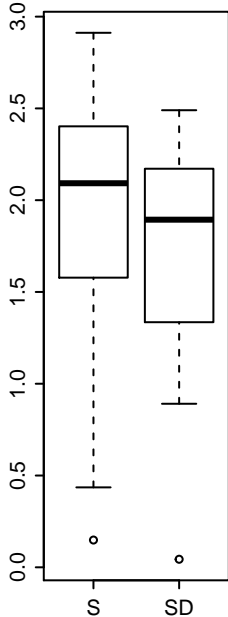

**Chao1**  
**p = 0.8374**

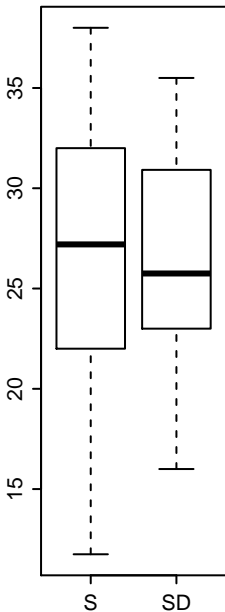

**Evenness**  
**p = 0.3572**

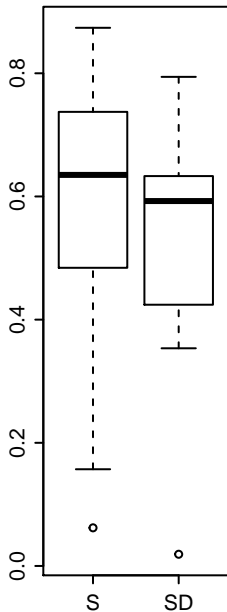

Supplement: S3 Fig — Each p-values obtained through the analysis of variance (ANOVA) was reported along with the name of the index analyzed. Boxes denote the interquartile range (IQR) between the 25th and the 75th percentile (first and third quartiles), whereas the inner line represents the median. For a more detailed description about boxes see Fig 1. (PDF) [file pone.0156807.s005.pdf]
