# Supplementary material for: Pyrosequencing Unveils Cystic Fibrosis Lung Microbiome Differences Associated with a Severe Lung Function Decline
Source: PLoS One. 2016 Jun 29;11(6):e0156807. doi: 10.1371/journal.pone.0156807 (PMC4927098; doi:10.1371/journal.pone.0156807)
Supplement: S1 Table — (DOCX) [file pone.0156807.s006.docx]

**S1 Table. Primers and barcodes used for 16S rRNA gene amplifications and sequencing.**

| Name | Sequence |
| --- | --- |
| A-adaptor | 5′-CCATCTCATCCCTGCGTGTCTCCGACTCAG-3′ |
| B-adaptor | 5′-CCTATCCCCTGTGTGCCTTGGCAGTCTCAG-3′ |
| 357F | 5′-CCTACGGGAGGCAGCAG-3′ |
| 926R | 5′-CCGTCAATTCMTTTRAGT-3′ |
| MID-1 | 5′-AAGCCGC-3′ |
| MID-2 | 5′-CAAGAAC-3′ |
| MID-3 | 5′-AGTTGGC-3′ |
| MID-4 | 5′-TATCAAC-3′ |
| MID-5 | 5′-AGGCGGC-3′ |
| MID-6 | 5′-CGGTATC-3′ |
| MID-7 | 5′-TGACGAC-3′ |
| MID-8 | 5′-ACAAGGC-3′ |
| MID-9 | 5′-AGACCTC-3′ |
| MID-10 | 5′-ATACCAC-3′ |
| MID-11 | 5′-TCGCGGC-3′ |
| MID-12 | 5′-ATCTTAC-3′ |
| MID-13 | 5′-AACCAGC-3′ |
| MID-14 | 5′-TTCGAGC-3′ |
| MID-15 | 5′-AAGGTGC-3′ |
| MID-16 | 5′-TCTTGGC-3′ |
| MID-17 | 5′-TAATCTC-3′ |
| MID-18 | 5′-TCACCTC-3′ |

Primers were designed linking B-adaptor to the 357F primer and A-adaptor to a barcode sequence and the 926R primer.
