# Supplementary material for: Pyrosequencing Unveils Cystic Fibrosis Lung Microbiome Differences Associated with a Severe Lung Function Decline
Source: PLoS One. 2016 Jun 29;11(6):e0156807. doi: 10.1371/journal.pone.0156807 (PMC4927098; doi:10.1371/journal.pone.0156807)
Supplement: S2 Table — (DOCX) [file pone.0156807.s007.docx]

**S2 Table. Number of sequences, OTUs, diversity indices and Good’s coverage estimator for all patients enrolled in the study.**

|  | Number of clones | Number of OTUs | Number of singletons | Number of doubletons | Chao 1 richness | Shannon diversity | Evenness | Good's coveragerage | Group | FEV_1_ |
| --- | --- | --- | --- | --- | --- | --- | --- | --- | --- | --- |
| BS1 | 2595 | 26 | 4 | 1 | 29.00 | 2.40 | 0.74 | 99.85 | S | I |
| BS2 | 2733 | 31 | 3 | 1 | 32.50 | 2.55 | 0.74 | 99.89 | S | I |
| BS4 | 2676 | 28 | 1 | 1 | 28.00 | 2.91 | 0.87 | 99.96 | S | I |
| BS32 | 1305 | 17 | 1 | 0 | 17.00 | 2.09 | 0.74 | 99.92 | S | I |
| BS33 | 2295 | 32 | 1 | 3 | 32.00 | 2.01 | 0.58 | 99.96 | S | I |
| BS7 | 3267 | 33 | 6 | 2 | 38.00 | 2.13 | 0.61 | 99.82 | S | I |
| BS17 | 2598 | 14 | 5 | 1 | 19.00 | 1.16 | 0.44 | 99.81 | S | I |
| BS29 | 5790 | 28 | 3 | 3 | 28.75 | 2.16 | 0.65 | 99.95 | S | I |
| GS2 | 3903 | 24 | 2 | 1 | 24.50 | 2.36 | 0.74 | 99.95 | S | I |
| GS1 | 3998 | 35 | 1 | 2 | 35.00 | 2.45 | 0.69 | 99.97 | S | I |
| GS4 | 1600 | 28 | 3 | 1 | 29.50 | 2.43 | 0.73 | 99.81 | S | I |
| GS3 | 2106 | 34 | 5 | 4 | 36.00 | 2.75 | 0.78 | 99.76 | S | I |
| MS1 | 880 | 19 | 4 | 3 | 20.50 | 1.32 | 0.45 | 99.55 | S | I |
| BS21 | 3859 | 11 | 3 | 3 | 11.75 | 1.09 | 0.45 | 99.92 | S | II |
| BS24 | 3205 | 17 | 2 | 0 | 18.00 | 1.31 | 0.46 | 99.94 | S | II |
| BS30 | 2126 | 27 | 2 | 4 | 27.20 | 1.60 | 0.48 | 99.91 | S | II |
| GS10 | 3306 | 27 | 2 | 2 | 27.33 | 1.80 | 0.55 | 99.94 | S | II |
| GS11 | 1671 | 29 | 5 | 1 | 34.00 | 2.27 | 0.67 | 99.70 | S | II |
| MS7 | 1158 | 20 | 3 | 0 | 23.00 | 1.62 | 0.54 | 99.74 | S | II |
| MS4 | 2970 | 19 | 4 | 1 | 22.00 | 1.92 | 0.65 | 99.87 | S | II |
| MS5 | 2096 | 16 | 6 | 1 | 23.50 | 0.44 | 0.16 | 99.71 | S | II |
| MS6 | 3306 | 11 | 3 | 0 | 14.00 | 0.15 | 0.06 | 99.91 | S | II |
| MS3 | 5765 | 22 | 4 | 2 | 24.00 | 0.98 | 0.32 | 99.93 | S | II |
| BS19 | 2427 | 23 | 2 | 2 | 23.33 | 1.81 | 0.58 | 99.92 | S | III |
| BS25 | 5288 | 35 | 3 | 0 | 38.00 | 2.79 | 0.79 | 99.94 | S | III |
| BS3R1 | 3318 | 12 | 0 | 1 | 12.00 | 1.58 | 0.64 | 100.00 | S | III |
| GS14 | 3710 | 31 | 2 | 4 | 31.20 | 2.14 | 0.62 | 99.95 | S | III |
| BS39 | 1989 | 32 | 1 | 3 | 32.00 | 2.60 | 0.75 | 99.95 | S | III |
| MS10 | 2767 | 22 | 2 | 2 | 22.33 | 2.09 | 0.68 | 99.93 | S | III |
| BNR9R1 | 3883 | 29 | 5 | 2 | 32.33 | 1.89 | 0.56 | 99.87 | SD | I |
| BNR10 | 4244 | 23 | 0 | 0 | 23.00 | 2.49 | 0.79 | 100.00 | SD | I |
| BNR13 | 3509 | 22 | 4 | 0 | 28.00 | 1.25 | 0.40 | 99.89 | SD | I |
| BNR22 | 2012 | 28 | 3 | 1 | 29.50 | 2.01 | 0.60 | 99.85 | SD | I |
| BNR28 | 1519 | 24 | 7 | 1 | 34.50 | 1.17 | 0.37 | 99.54 | SD | I |
| GNR5 | 3814 | 35 | 2 | 1 | 35.50 | 2.16 | 0.61 | 99.95 | SD | I |
| GNR6 | 4150 | 34 | 2 | 2 | 34.33 | 2.18 | 0.62 | 99.95 | SD | I |
| GNR7 | 3255 | 30 | 5 | 1 | 35.00 | 1.54 | 0.45 | 99.85 | SD | I |
| GNR9 | 3095 | 23 | 1 | 0 | 23.00 | 2.45 | 0.78 | 99.97 | SD | I |
| MNR2 | 1851 | 25 | 6 | 3 | 28.75 | 1.43 | 0.44 | 99.68 | SD | I |
| BNR11 | 2383 | 14 | 3 | 0 | 17.00 | 1.56 | 0.59 | 99.87 | SD | II |
| BNR12 | 3741 | 23 | 1 | 1 | 23.00 | 2.46 | 0.78 | 99.97 | SD | II |
| BNR16 | 3095 | 10 | 4 | 0 | 16.00 | 0.89 | 0.39 | 99.87 | SD | II |
| BNR23 | 3161 | 17 | 3 | 3 | 17.75 | 1.15 | 0.41 | 99.91 | SD | II |
| GNR12 | 3959 | 25 | 1 | 0 | 25.00 | 1.78 | 0.55 | 99.97 | SD | II |
| GNR13 | 1506 | 25 | 3 | 3 | 25.75 | 2.19 | 0.68 | 99.80 | SD | II |
| MNR9 | 2276 | 34 | 2 | 1 | 34.50 | 2.33 | 0.66 | 99.91 | SD | II |
| BNR18R1 | 1878 | 16 | 2 | 2 | 16.33 | 0.98 | 0.35 | 99.89 | SD | III |
| BNR15 | 4044 | 10 | 6 | 0 | 25.00 | 0.04 | 0.02 | 99.85 | SD | III |
| BNR20 | 2787 | 13 | 4 | 0 | 19.00 | 1.58 | 0.62 | 99.86 | SD | III |
| BNR37 | 6381 | 25 | 0 | 1 | 25.00 | 2.08 | 0.65 | 100.00 | SD | III |
| GNR15 | 3747 | 29 | 2 | 1 | 29.50 | 2.04 | 0.61 | 99.95 | SD | III |
| BNR38 | 3110 | 26 | 4 | 1 | 29.00 | 1.93 | 0.59 | 99.87 | SD | III |

Number of clones is the number of sequences correctly assigned to a particular sample. The number of sequences and OTUs assigned to each patient has been reported after the sequence-processing step.

The number of OTUs correspond to richness of the given sample, whereas the number of singletons and doubletons is the number of sequences found only 1 or 2 times, respectively. S: stables; SD: sever decliners.
