# Supplementary material for: Pyrosequencing Unveils Cystic Fibrosis Lung Microbiome Differences Associated with a Severe Lung Function Decline
Source: PLoS One. 2016 Jun 29;11(6):e0156807. doi: 10.1371/journal.pone.0156807 (PMC4927098; doi:10.1371/journal.pone.0156807)
Supplement: S3 Table — (DOCX) [file pone.0156807.s008.docx]

**S3 Table. Multivariate analysis of variance (MANOVA) on OTUs dataset.**

|  | Df | Pillai | approx F | num Df | den Df | Pr(>F) |  |
| --- | --- | --- | --- | --- | --- | --- | --- |
| FEV_1_ | 2 | 1.92801 | 2.4346 | 88 | 8 | 0.08837 |  |
| Condition | 1 | 0.99233 | 8.8228 | 44 | 3 | 0.04816 | * |
| FEV_1_:Condition | 2 | 1.87255 | 1.3357 | 88 | 8 | 0.35153 |  |
| Residuals | 46 |  |  |  |  |  |  |

Data are presented reporting the two factors used in the analysis (FEV_1_ and Condition) and the interaction between them (FEV_1_:Condition). Df: degrees of freedom; Pillai: Pillai–Bartlett statistic; approx. F: F distribution statistic; num Df: degrees of freedom of the numerator; den Df: degrees of freedom of the denominator; Pr(>F): two-tailed significance probability.
